# MicroRNA-17-92 significantly enhances radioresistance in human mantle cell lymphoma cells

**DOI:** 10.1186/1748-717X-5-100

**Published:** 2010-11-01

**Authors:** Ping Jiang, En Y Rao, Na Meng, Yong Zhao, Jun J Wang

**Affiliations:** 1 Department of Radiation Oncology, Peking University Third Hospital, Beijing 100191, China; 2 Transplantation Biology Research Division, State Key Laboratory of Biomembrane and Membrane Biotechnology, Institute of Zoology, Chinese Academy of Sciences, Beijing 100101, China

## Abstract

The microRNA-17-92 (miRNA-17-92) cluster, at chromosome 13q31-q32, also known as oncomir-1, consists of seven miRNAs that are transcribed as a polycistronic unit. Over-expression of miRNA-17-92 has been observed in lymphomas and other solid tumors. Whether miRNA-17-92 expression affects the response of tumor cells to radiotherapy is not addressed so far. In the present study, we studied the effects of miRNA-17-92 on the radiosensitivity of human mantle cell lymphoma (MCL) cells Z138c. Over-expression of miRNA-17-92 significantly increased survival cell number, cell proliferation and decreased cell death of human MCL cells after different doses of radiation. Immunoblot analysis showed that phosphatase and tension homolog (PTEN) and PHLPP2 was down-modulated and pAkt activity was enhanced in MCL cells after over-expressing miRNA-17-92 after irradiation. These findings are the first direct evidence that over-expression of miRNA-17-92 cluster significantly increases the radioresistance of human MCL cells, which offers a novel target molecule for improving the radiotherapy of MCL in clinic.

## Introduction

The importance of microRNAs in cancer is highlighted by the observation that approximately 50% of miRNA genes are located in cancer-associated genomic regions or in fragile sites [1,2], which are frequently amplified or deleted in tumorigenesis. Mantle cell lymphoma (MCL) is an aggressive hematological malignancy, characterized by the chromosomal translocation t(11;14)(q13;q32), which results in deregulated aberrant expression of cyclin D1, and comprises 5%-10% of human B-cell malignancies [3]. The median survival of patients with MCL ranges between 3 and 5 years according to most studies [4,5]. Studies in transgenic mice imply that the t(11;14)(q13;q32) translocation alone is not sufficient to result in lymphoma, and additional genetic alterations are necessary [6,7]. Secondary genomic alterations are frequently detected in MCL, of which chromosome 13q31-q32 gain/amplification is one of the most frequent [8,9]. Studies have shown that amplification at chromosome 13q31-q32 targets a microRNA cluster, microRNA-17-92 (miRNA-17-92), which resides within intron 3 of c13orf25, a non-protein-coding gene at 13q31.3 [10,11].

The miRNA-17-92 cluster, which modulates E2F1 expression, is positively regulated by MyC [12], can potentially become a very potent oncogene, targeting multiple cellular pathways and favoring tumorigenesis by enhancing cell proliferation and inhibiting apoptosis. Previous data have shown that miRNA-17-92 can increase MyC-enhanced proliferation by targeting p21 and consequently activating the cyclinD1/CDK4 complex to release retinoblastoma inhibition of E2F genes [13,14]. miRNA-17-92 is also capable of minimizing MyC-induced apoptosis by targeting the Bcl2-like Bim and phosphatase and tension homolog (PTEN) genes [15] to increase the level of anti-apoptotic BCL2.

Radiation therapy is one of the three primary modalities used in cancer treatment. Whether miRNA-17-92 expression affects the response of tumor cells to radiotherapy has not been investigated so far. To elucidate this issue, we generated stable MCL cell lines with high expression of the miRNA-17-92 cluster and the radiosensitivity was determined. We found that over-expression of miRNA-17-92 in MCL cells remarkably decreases the radiosensitivity of the MCL cell line Z138c while the activity of PI3K/Akt pathway is enhanced possibly via down-regulation of PTEN and PHLPP2. We thus offered first evidence that miRNA17-92 is closely involved in the radioresistance of tumor cells.

## Materials and methods

### Plasmid, cell lines and cell transfection

The tetracyclin-regulated retroviral vector TMP (OpenBioSystem, Huntsville, AL) was modified by deleting the miR-30 sequence using PCR with the following primers: 5'-PO4-GCCTCGAGCCTGAGGCTGGATCGGTCCCGGTGTCTTCTATGG-3', and 5'-PO4-TGAGGGAATTCGGACCGGGTAGGGGAGGCGCTTTTCCCAAG-3'. The PCR product was then circularized by blunt-end ligation to generate the miRNA-17-92 cluster was amplified from human genomic DNA using the following primers: 5'-tttttctcgaGTGTCTAAATGGACCTCATATCTTTGAG-3', and 5'-gtttttgaattCCAAATCTGACACGCAACCC-3' (antisense) and Phusion Taq Polymerase (New England Biolabs, Boston, MA). The PCR product was then cloned into the TMP2 vector to generate the plasmid TMP2-miR-17-92. Vector TMP2 and plasmid TMP2-miR-17-92 were kindly provided by Dr En Y Rao who was in Institute of Zoology, Chinese Academy of Sciences. To construct 3'untranslated region (UTR) luciferase reporter plasmids, the pGL3 vector with luciferase coding sequence purchased from Promega company, USA.

The expression level of mature miRNAs was determined using the TaqMan miRNA Assay (Applied Biosystems, Foster City, CA) with slight modification. Briefly, single-stranded cDNA was synthesized from 10 ng of total RNA using the TaqMan MicroRNA Reverse Transcription Kit. Each cDNA generated was amplified by quantitative PCR using sequence-specific primers from the TaqMan MicroRNA Assays (Human Panel) on a 7900HT Sequence Detection System. The relative quantity of the target miRNAs was estimated by the 2^-∆∆CT ^method by normalizing to the expression level of β-actin, which was detected by a TaqMan gene expression Assay.

Human mantle cell lymphoma (MCL) cell line Z138c was provided by institute of zoology, Chinese Academy of Sciences.

Tetracycline-regulated pRevTet-On expression system purchased from Clontech, USA, operated according to the manufacturer's instructions. The human embryonic kidney cell line HEK293T was co-transfected with the pRevTet-On vector and pCL packaging plasmid using the calcium phosphate method. The virus supernatant was collected and used to infect Z138c. The transfected cell line Z138c-Tet-On was selected with G418 (1 μg/ml) which purchased from Sigma company USA. To further establish TMP2-miR17-92 cell line, the HEK293T cell line was co-transfected with the TMP2-miR-17-92 vector and pCL packaging plasmid by the calcium phosphate method, and the virus supernatant was collected and used to transfect the established Z138c-Tet-on cells. These cells were further selected with puromycin resistance and green fluorescent protein (GFP) expressing cells were isolated by fluorescence activated cell sorter, (FACS). The cell lines which overexpress miR-17-92 were maintained in the presence of doxycycline (1 μg/ml).

The HEK293T cell line was co-transfected with the TMP2 vector and pCL packaging plasmid by the calcium phosphate method, and the virus supernatant was collected and used to infect the established Z138c-Tet-on cells. And then Z138c-TMP2 cell line was generated.

### Cell culture

The Z138c-miRNA-17-92 cell lines and Z138c-TMP2 cell lines were suspended in RPMI1640 supplemented with 10% fetal bovine serum (FBS), 100 UI/ml penicillin, and 100 UI/ml streptomycin. Doxycycline (1 μg/ml) was added to induce the expression of miRNA-17-92. The cells were incubated in a humidified atmosphere of 5% CO_2 _at 37°.

### Irradiation conditions

Linear accelerators producing 6 MV X-ray beams were provided by the 306 Hospital of the People's Liberation Army (Beijing, China). The dose rate was 400 cGy/min and the source-to-skin distance (SSD) was 100 cm. The surface of the culture dishes was covered by 2 cm of packing materials. Radiation doses were: 0, 0.5, 1, 2, 3, 4 and 6 Gy.

### Viable cell count

Z138c-TMP2 and Z138c-TMP2-miRNA-17-92 cells in exponential growth were irradiated by 6 MV X-ray at various doses (0, 0.5, 1, 2, 3, 4, and 5 Gy). Three wells in each dose in 24 well plates were cultured for 24, 48, 72 or 96 h in an incubator, cells were stained with trypan blue for the viable count estimation.

### Cell proliferation measured by ^3^H-TdR incorporation

Z138c-TMP2 and Z138c-miRNA-17-92 cells in exponential growth were irradiated by 6 MV X-ray at various doses (0, 0.5, 1, 2, 3, 4, 5, and 6 Gy). Cells were plated in 96-well plates in 200 μl of growth medium and allowed to attach for 12, 36, 60, 84 or 112 h, and 0.5 μCi ^3^H-TdR per well was added respectively, then incubated for 12 h at 37°. After incubation the cells were collected and distributed through a glass fiber filter by using a multiple head cell harvester type DYQ-Ⅱ. When the filter membrane was dry, the corresponding membranes were cut off and put into 5 ml of scintillation solution to be detected.

### Cell cycle analysis by flow cytometry (FCM)

Z138c-TMP2 and Z138c-miRNA-17-92 cells in exponential growth were irradiated by 6 MV X-ray at various doses (0, 2, and 4 Gy). 24 h after irradiation, cell cycle was analyzed using FCM as described previously.

Z138c-TMP2 and Z138c-TMP2-miRNA-17-92 cells in exponential growth were irradiated by 6 MV X ray at various doses (0, 2, and 4 Gy). 24 h after irradiation, 2 × 10^6 ^cells were taken from each sample to be tested, which were fixed by 3 ml 70% alcohol at -20° over night. Then washed by PBS twice and suspended in 200 ul PBS, placed in 37° water for 30 min, mixed with 10 mg/ml RNA enzyme and 500 μg/ml PI, waiting to be tested.

### Apoptosis and necrosis analysis by FCM

Z138c-TMP2 and Z138c-TMP2-miRNA-17-92 cells in exponential growth were irradiated by 6 MV X ray at various doses (0, 2, and 4 Gy, respectively). 72 hours later, cells were plated in 24-well plates and 1 ml 1% Hochest33324 per well was added respectively, taken photo after 30~40 min.

Z138c-TMP2 and Z138c-TMP2-miRNA-17-92 cells in exponential growth were irradiated by 6 MV X ray at various doses (0, 2, and 4 Gy, respectively). 72, 96, 120 hours after irradiation, the cells were stained by Propidium iodide (PI) to detect the percent of cell death.

### Immunoblotting and antibodies

Cell lysates containing 20 μg of protein were resolved on sodium dodecyl-sulfate polyacrylamide gel electrophoresis (SDS-PAGE) and transferred to nitrocellulose membranes (Hybond-P, Amersham, Buckinghamshire, UK). The membranes were incubated with 5% non-fat milk blocking buffer (TBS-T) for 1 h at room temperature and then incubated overnight at 4°C with the primary antibodies. Membranes were washed with PBS containing 0.1% Tween-20 (PBS-T), then incubated in the dark for 1 h at room temperature with IRDye 680-conjugated goat anti-rabbit IgG or IRDye 800 conjugated goat anti-mouse IgG in Odyssey blocking buffer. After washing with PBS-T, proteins were detected and quantified using the Odyssey Infrared Imaging System (LI-COR Biosciences). For each study, data were representative of three independent experiments.

Antibodies for immunoblotting in this study were as follows: anti-PTEN, anti-Akt, anti-p-Akt-ser473 (Cell Signaling Biotechnology, Beverly, MA, USA), anti-goat IgG-HRP, anti-actin, and anti-PH domain leucine-rich repeat protein phosphatase (PHLPP) (Novus Biologicals, Littleton, CO, USA).

### Statistical analysis

All data have been presented as the mean ± s.d. Student's unpaired t-test for comparison of means has been used to compare groups. A P-value 0.05 has been considered to be statistically significant.

## Results

### The over-expression of miRNA-17-92 significantly enhanced survival of Z138c cells after different doses of radiation

**Figure 1:**
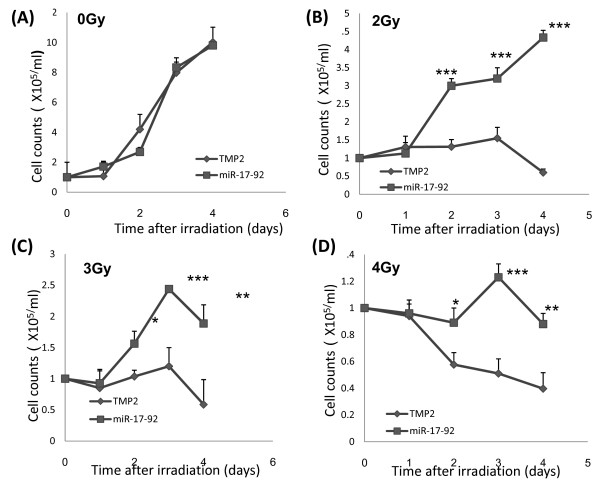
**The over-expression of miRNA-17-92 significantly enhanced survival of Z138c cells after different doses of radiation**. Z138c-TMP2 or Z138c-miRNA-17-92 cells were cultured for different days after receiving 0 (**A**), 2 (**B**), 3 (**C**), or 4 (**D**) Gy X-ray irradiation. Data have been presented as mean ± s.d. (N = 5). One representative of three experiments has been shown. *P < 0.05, **P < 0.01 or ***P < 0.001 as compared among the identical groups.

In order to determine whether over-expression of miRNA-17-92 could change the survival of Z138c cells after ionizing irradiation, we counted the viable cells after different doses of irradiation and at different time points. As shown in Figure 1, there were no differences between the two groups in viable Z138c-TMP2 or Z138c-miRNA-17-92 cell counts without radiation. However, viable cell counts were significantly higher in the miRNA-17-92 group than in the TMP2 group when these cells received different doses of irradiation by 1 day after irradiation (P < 0.05, P < 0.01, and P < 0.001, respectively, Figure 1).

### The different proliferative ability of Z138c-TMP2 and Z138c-miRNA-17-92 cells after different doses of radiation

**Figure 2:**
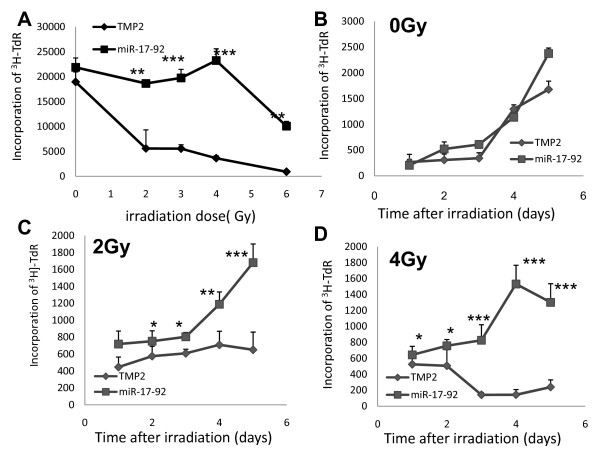
**The different proliferative ability of Z138c-TMP2 and Z138c-miRNA-17-92 cell lines after different doses of radiation**. The cell proliferation of Z138c cells expressing miRNA-17-92 or control vector was detected using a ^3^H-TdR incorporation assay after different doses of irradiation. **A**) the cell proliferation of Z138c-TMP2 or Z138c-miRNA-17-92 cells after different doses of radiation. **B**) the cell proliferation of cells without radiation. **C**) the cell proliferation of Z138c-TMP2 or Z138c-miRNA-17-92 cells at different days after 2 Gy radiation. **D**) the cell proliferation of cells at different days after 4 Gy radiation. Data have been presented as mean ± s.d. (N = 5). One representative of three experiments has been shown. *P < 0.05, **P < 0.01 or ***P < 0.001 as compared among the identical groups.

To investigate the effect of miRNA-17-92 on the proliferating ability of tumor cells, we detected the cell proliferation of Z138c cells expressing miRNA17-92 or control vector after irradiation using a ^3^H-TdR incorporation assay. There were no difference between the two groups after radiation at 0 Gy (Figure 2). However, statistically significant differences were obtained at a radiation dose of 2 Gy and incubation times of 48, 72, 96, and 120 h and at a radiation dose of 4 Gy and incubation times of 24, 48 h, 72, 96, and 120 h (P < 0.05, P < 0.01 or P < 0.001, Figure 2).

### The cell cycle distribution of Z138c-TMP2 and Z138c-miRNA-17-92s cells after different doses of radiation

**Figure 3:**
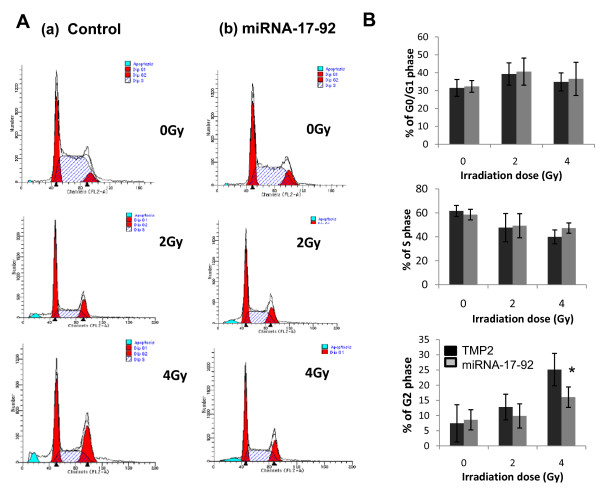
**The cell cycle distribution of Z138c-TMP2 and Z138c-miRNA-17-92 cells after different doses of radiation**. The cell cycle of Z138c-TMP2 and Z138c-miRNA-17-92 cells was determined by PI staining and detected by FCM at 1 day after radiation. **A**) one representative of cell cycle distribution as detected by FCM. **B**) a summary of different cell phases of Z138c-TMP2 and Z138c-miRNA-17-92 cell lines. Data have been presented as mean ± s.d. (N = 5). One representative of three experiments has been shown. *P < 0.05 as compared among the identical groups.

The cell cycle was determined by PI staining and assayed by FCM. The percentage of G2/M cells in the Z138c-TMP2 cells increased after radiation doses of 2 Gy and 4 Gy comparing with the non-irradiated cells (Figure 3). However, no obvious radiation-induced G2/M cell cycle arrest was observed in Z138c-miRNA-17-92 cells. A statistically significant difference (t = 2.885, P < 0.05) was obtained at a radiation dose of 4 Gy compared between Z138c-TMP2 and Z138c-miRNA-17-92 cells.

### The cell death of Z138c-TMP2 and Z138c-miRNA-17-92s cells after different doses of radiation

**Figure 4:**
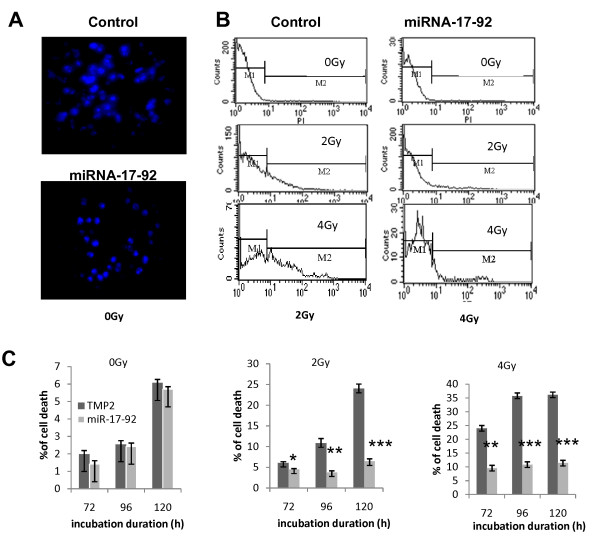
**The cell death of Z138c-TMP2 and Z138c-miRNA-17-92s after different doses of radiation**. The cell death ratios of Z138c-TMP2 cells and Z138c-miRNA-17-92 cells were evaluated using traditional PI staining assay. **A**) one representative of cell death as detected by 1% Hochest33324 staining at 3 days after radiation. **B**) one representative of cell death as detected by FCM at 3 days after radiation. **C**) a summary of cell death ratios in Z138c-TMP2 and Z138c-miRNA-17-92 cells. Data have been presented as mean ± s.d. (N = 5). One representative of three experiments has been shown. *P < 0.05, **P < 0.01 or ***P < 0.001 as compared among the identical groups.

The cell death ratio was evaluated using traditional PI staining assay. As shown in Figure 4, more dead cells were seen in the Z138-TMP2 cells than in Z138c-miRNA-17-92 cells at 72, 96, and 120 h after radiation, regardless of radiation doses, respectively. Statistically significant differences were observed at a radiation dose of 2 Gy and incubation times of 96, or 120 h and at a radiation dose of 4 Gy and incubation times of 96 or 120 h (P < 0.05, P < 0.01 or P < 0.001, respectively, Figure 4).

### The expression of the proteins pAkt, PTEN and PHLPP2 in Z138c-TMP2 and Z138c-miRNA-17-92 cells after radiation

**Figure 5:**
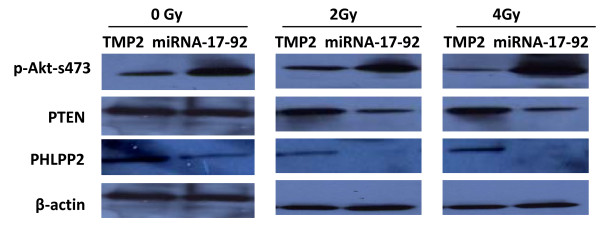
**The expression of the proteins p-Akt, PTEN and PHLPP2 in Z138c-TMP2 and Z138c-miRNA-17-92 cells after radiation**. The protein expression of pAkt, PTEN and PHLPP2 in both cell lines was detected by immunoblot analysis at 1 day after radiation as described in materials and methods. One representative of three experiments has been shown.

As PTEN and PHLPP2 are the target genes of miRNA-17-92, we thus examined the protein expression of pAkt, PTEN and PHLPP2 in both cell lines by immunoblot analysis after radiation. As shown in Figure 5, compared with Z138c control cells, PTEN and PHLPP2 protein levels were reduced in Z138c-miRNA-17-92 cells after radiation. Consistently, pAkt was enhanced in Z138c-miRNA-17-92 cells after radiation.

## Discussion

MCL is considered incurable with the current chemotherapeutic regimen. Gene expression profiling (GEP) studies have shown that the survival of MCL patients is closely correlated with the proliferation signature of the tumor cells [16]. It is interesting that over-expression of c13orf25, the primary transcript from which miRNA-17-92 is processed, has been associated with increased expression of genes associated with proliferation and poorer survival. Since this observation is based on GEP analysis, further large-scale, confirmatory, clinical studies using more specific approaches are warranted. In the present study, we demonstrated that over-expression of miRNA17-92 in tumor cells can significantly enhance the resistance to radiation-induced cell damage including cell death and G2/M phase arrest. Our findings suggest that targeting the miRNA-17-92 cluster may provide a novel therapeutic approach for MCL patients. It may be important for us to see whether the expression of this cluster is closely relevant to the radiation sensitivity or not in the clinical cases in the future.

PTEN is a lipid phosphatase that removes the activating signal and ultimately prevents Akt phosphorylation and activation, while PHLPP2 terminates Akt signaling by directly dephosphorylating and inactivating Akt, thus, both pTEN and PHLLP2 negatively regulates PI3K/Akt signaling pathway, which is one of the most important pathways for cell survival and inhibition of apoptosis [17-20]. Deletion of the chromosome 10 PTEN gene plays a role in tumor suppression. After X-ray radiation, Z138c cells with over-expression of miRNA-17-92 showed down-modulated tumor suppressors PTEN and PHLPP2 and enhanced pAkT as determined by western blot. Ramaswamy et al [21] showed that suppression of the PTEN/PI3K/AKT signaling pathway may increase the radiosensitivity of malignant brain neurogliocytoma cells. Our present study showed that over-expression of miRNA-17-92 decreased both PTEN and PHLPP expression and thereby enhanced PI3K pathway and finally results in cell death resistance induced by x-rays. Because of the complexity of miRNA-17-92 function, more targets may exist for regulating the cell transduction signal by miRNA-17-92 through various modes, which will be a future goal of our research.

In a summary, miRNA-17-92 is closely involved in the regulation of radiosensitivity of tumor cells. It directly down-regulates the expression of the PTEN and PHLPP2 proteins, subsequently activates the PI3K/Akt signal pathway, and thus results in the resistance to radiation of the MCL Z138c cell line. MiRNA-17-92 may be a potential molecular target for improving the radiotherapy.

## Competing interests

The authors declare that they have no competing interests.

## Authors' contributions

PJ carried out cell colony-forming assay, fluorescence-activated cell sorting, flow cytometric analysis, and drafted the manuscript. JJW participated in its design and revised the manuscript. NM performed the statistical analysis and carried out the irradiation experiment. YZ and EYR supervised experimental work and revised the manuscript. All authors read and approved the final manuscript.
